# Transfer of a mobile *Staphylococcus saprophyticus* plasmid isolated from fermented seafood that confers tetracycline resistance

**DOI:** 10.1371/journal.pone.0213289

**Published:** 2019-02-28

**Authors:** Jong-Hoon Lee, Sojeong Heo, Miran Jeong, Do-Won Jeong

**Affiliations:** 1 Department of Food Science and Biotechnology, Kyonggi University, Suwon, Republic of Korea; 2 Department of Food and Nutrition, Dongduk Women’s University, Seoul, Republic of Korea; Korea University, REPUBLIC OF KOREA

## Abstract

The complete nucleotide sequence of a tetracycline-resistance gene (*tetK*)-carrying plasmid from a *Staphylococcus saprophyticus* isolate from jeotgal, a Korean high-salt-fermented seafood, was determined. The plasmid, designated pSSTET1, was 4439 bp in length and encoded typical elements found in plasmids that replicate via a rolling-circle mechanism, including the replication protein gene (*rep*), a double-stranded origin of replication, a single-stranded origin of replication, and a counter-transcribed RNA sequence. Additionally, the plasmid recombination enzyme gene (*pre*), which may be involved in inter-plasmid recombination and conjugation, was found. Each gene exhibited >94% sequence identity with those harbored in other *Staphylococcus* species. pSSTET1 was conditionally transferred to *Staphylococcus* species in a host-dependent manner and transferred to an *Enterococcus faecalis* strain *in vitro*. Antibiotic susceptibility of the transconjugants was host-dependent and transconjugants maintained a tetracycline-resistant phenotype in the absence of selective pressure over 100 generations.

## Introduction

For decades, antibiotic resistance studies of bacteria have mainly focused on clinically-important species that are directly exposed to antibiotics; however, antibiotic-resistant bacteria are found in diverse niches including soil, water, foods, and the gastrointestinal tract. Recently, many studies have speculated that commensal bacteria may act as reservoirs of antibiotic resistance genes that can be transferred to other resident intestinal bacteria or transient bacteria that pass through [1]. The intestinal microflora is a potential source of antibiotic-resistant pathogens and the food chain is considered as one of the possible transfer routes of antibiotic resistance from animal and environment-associated antibiotic-resistant bacteria into the human gastrointestinal tract where these genes may be transferred to pathogenic and opportunistic bacteria [2, 3]. In this context, fermented and raw foods harboring large numbers of living bacteria have received increasing attention as potential vehicles of antibiotic resistance determinants [4], which can be transferred to the gut microbiota.

In our cultivable bacterial community analysis of two types of jeotgal, a Korean high-salt-fermented seafood, coagulase-negative staphylococci (CNS) was identified as a predominant bacterial group [5]. The following safety and technological property assessments of CNS isolates to select safe and efficient starter candidates, a number of isolates exhibiting resistance to one or more antibiotics were identified [6, 7]. Among the antibiotic-resistant CNS isolates, a *Staphylococcus saprophyticus* strain KM1053 exhibited resistance to penicillin G and tetracycline [6, 7]. A known *tetK*-specific PCR primer set successfully amplified a partial tetracycline efflux protein gene *tetK* from strain KM1053 [8]. The detection of tetracycline-resistant bacteria in fermented food warrants special attention because tetracycline is the first-line treatment for a number of infections in many parts of the world [9] and tetracycline resistance in most bacteria is acquired through new genes, often associated with mobile elements such as plasmids [10, 11]. Food-originated bacteria harboring a mobile tetracycline resistance gene can contribute to the spread of resistant genes to human microbiota and pose a risk to human health.

Despite several reports on the advent of tetracycline-resistant bacteria in fermented foods, studies on the transfer of tetracycline-resistant genes in food-associated bacteria, especially CNS, have been rarely reported [12, 13]. We characterized the *tetK*-carrying plasmid of *S*. *saprophyticus* strain KM1053 and illuminated its transferability between species involved in food fermentation to demonstrate the possibility of horizontal antibiotic resistance gene transfer within food matrices.

## Materials and methods

### Bacterial strains and cultures

CNS strains, *S*. *saprophyticus* KM1053, *Staphylococcus equorum* KM1031, and *S*. *equorum* KS1039 isolated from jeotgal and stored in our stock cultures, were used in the current study [5]. Strain KM1053 was used to characterize the *tetK*-encoded plasmid and to assess the *tetK*-encoded plasmid transferability as a donor strain. *S*. *saprophyticus* KM1053 has been deposited in the Korean Collection for Type Cultures under resource number KCTC 43017. Two *S*. *equorum* strains KM1031 and KS1039 used as recipient strains for plasmid transfer experiments have been deposited in the Korean Culture Center of Microorganisms under resource numbers KCCM 43181 and KCCM 43182, respectively (Table 1). *Staphylococcus aureus* USA300 LAC strain, a clinical isolate [14, 15], and *Enterococcus faecalis* OG1RF, a human isolate [16], were adopted as recipients for plasmid transfer experiments. All strains except *E*. *faecalis* OG1RF were primarily cultured in tryptic soy agar (TSA; BD Diagnostic Systems, Sparks, MD, USA) and tryptic soy broth (TSB; BD Diagnostic Systems) at 30 °C for 24 h to maintain their phenotypic traits. *E*. *faecalis* OG1RF traits were maintained in De Man-Rogosa-Sharpe (MRS; BD Diagnostic Systems) media at 30 °C for 24 h.

**Table 1:** Bacterial strains examined in this study and their corresponding MICs.

Strain	Origin	Phenotype	MIC (mg/l) against antibiotics				Reference
			Tet	Chl	Ery	Lin	
*S*. *saprophyticus* KM1053	Myeolchi-jeotgal	Tet^R^, Pen^R^	32	<0.5	<0.5	<0.5	[6]
*S*. *equorum* KM1031	Myeolchi-jeotgal	Ery^R^, Chl^R^, Lin^R^, Pen^R^	<0.5	128	32	1024	[36]
*S*. *equorum* KS1039	Saeu-jeotgal		<0.5	<0.5	<0.5	<0.5	[7, 50]
*S*. *aureus* USA300 LAC	Human	Amp^R^, Ery^R^	2	8	64	0.5	[14, 15]
*E*. *faecalis* OG1RF	Human	Lin^R^	<0.5	4	2	256	[16]

Strain KM1053 has been deposited in the Korean Collection for Type Cultures under resource number KCTC 43017. Strains KM1031 and KS1039 have been deposited in the Korean Culture Center of Microorganisms under resource numbers KCCM 43181 and KCCM 43182, respectively.

Abbreviations: Amp, ampicillin; Chl, chloramphenicol; Ery, erythromycin; Lin, lincomycin; Pen, penicillin G; Tet, tetracycline.

### Identification and sequence analysis of the *tetK*-carrying plasmid

Plasmid DNAs were extracted from strain KM1053 with a plasmid mini prep kit (Inclone Biotech, Daejeon, Korea) after lysostaphin (40 μg/ml) treatment at 37 °C for 30 min to lyse the cell walls. The plasmid DNAs were then used as templates for the PCR amplification of the *tetK*-carrying plasmid by gene walking. PCR primers complementary to the partially determined *tetK* gene were used to amplify its flanking regions and the full plasmid sequence was determined by PCR walking. PCR amplification was conducted over 30 cycles which included denaturing at 95 °C for 1 min, annealing at 58 °C for 1.5 min, and extending at 72 °C for 1 min with Ex *Taq* polymerase (Takara, Kyoto, Japan) using a T3000 Thermocycler (Biometra, Gottingen, Germany). PCR products were purified using a gel and PCR purification kit (Inclone Biotech). DNA sequences were determined by a custom service provided by Bionics (Seoul, Korea). DNA and amino acid sequence data analyses were performed using the Lasergene sequence analysis software package (Dnastar, Madison, USA). Sequence similarities were identified using the BLASTX program at the National Center for Biotechnology Information website (http://blast.ncbi.nlm.nih.gov/).

### Plasmid transfer experiment

To determine the transferability of the *tetK*-carrying plasmid, the *S*. *saprophyticus* KM1053 strain was mated with different recipient strains using the broth mating method [17]. Recipient strains were tetracycline sensitive and conferred specific antibiotic resistance to facilitate transconjugant selection. Logarithmic phase donor cells cultured in Mueller—Hinton (MH) broth (BD Diagnostic Systems) were mixed with logarithmic phase recipient cells cultured in MH broth at a 1:10 ratio and incubated at 30 °C for 3 h. The mixture was spread onto the surface of TSA plates supplemented with 10 mg/l tetracycline and other appropriate antibiotics. Other antibiotics were used at the following concentrations: erythromycin, 10 mg/l; and lincomycin, 30 mg/l. Transconjugants were selected after incubation at 30 °C for 24 h, and were confirmed by colony PCR with primers corresponding to *tetK* [7, 18]. Recipient traits of transconjugants were confirmed by 16S rRNA gene sequence analysis.

### Minimum inhibitory concentration (MIC) determination

Antibiotic MICs were determined by the broth microdilution method as described previously [19]. Each antibiotic was prepared with serial two-fold working dilutions in deionized water and the final concentration of each antibiotic in one 96-microwell plate ranged between 0.5 and 4096 mg/l. Bacterial strains were cultured twice in TSB and matched a McFarland 0.5 turbidity standard (bioMérieux, March L’Etoile, France). Each suspension was diluted a further 1:100 in cation-adjusted MH broth to achieve an adequate inoculum concentration. The final inoculum density was 5 × 10^5^ colony-forming units (cfu)/ml per well on 96-microwell plates. The MIC of each antibiotic was recorded as the lowest concentration where no growth was observed in the wells after incubation for 18 h or 24 h. MIC results were confirmed by at least three independently performed tests.

### Evaluation of plasmid stability

The segregational stability of the *tetK*-encoded plasmid in *S*. *saprophyticus* KM1053 and transconjugants was determined as described previously [20]. Briefly, a single colony was inoculated into selection-pressure-free TSB and cultured at 30 °C for 24 h. The saturated culture was diluted to 10^˗3^ in fresh TSB, and this same dilution was repeated into fresh TSB every 24 h. Each culture sample was spread on TSA plates following serial dilution and incubated overnight at 30 °C. Individual colonies were picked and streaked onto TSA plates containing 10 mg/l tetracycline to check for tetracycline resistance. Additionally, possession of pSSTET1 was confirmed by colony PCR with primers (P126: 5'- GTCACCTCAAGTAAAGAGG-3' and P190: 5'- CAGAGGGAACAGGTATAGC-3'), which can amplify the plasmid, and sequence analysis of the amplicon.

## Results

### Nucleotide sequence of the plasmid carrying *tetK*

The sequence of partially amplified *tetK* from tetracycline-resistant *S*. *saprophyticus* strain KM1053 was identical to that of the previously characterized *tetK* on pPM1 of *S*. *aureus* strain PM1 [21]. We successfully amplified and confirmed a plasmid of 4439 bp in size carrying *tetK* by PCR gene walking and designated it as pSSTET1.

**Fig 1:**
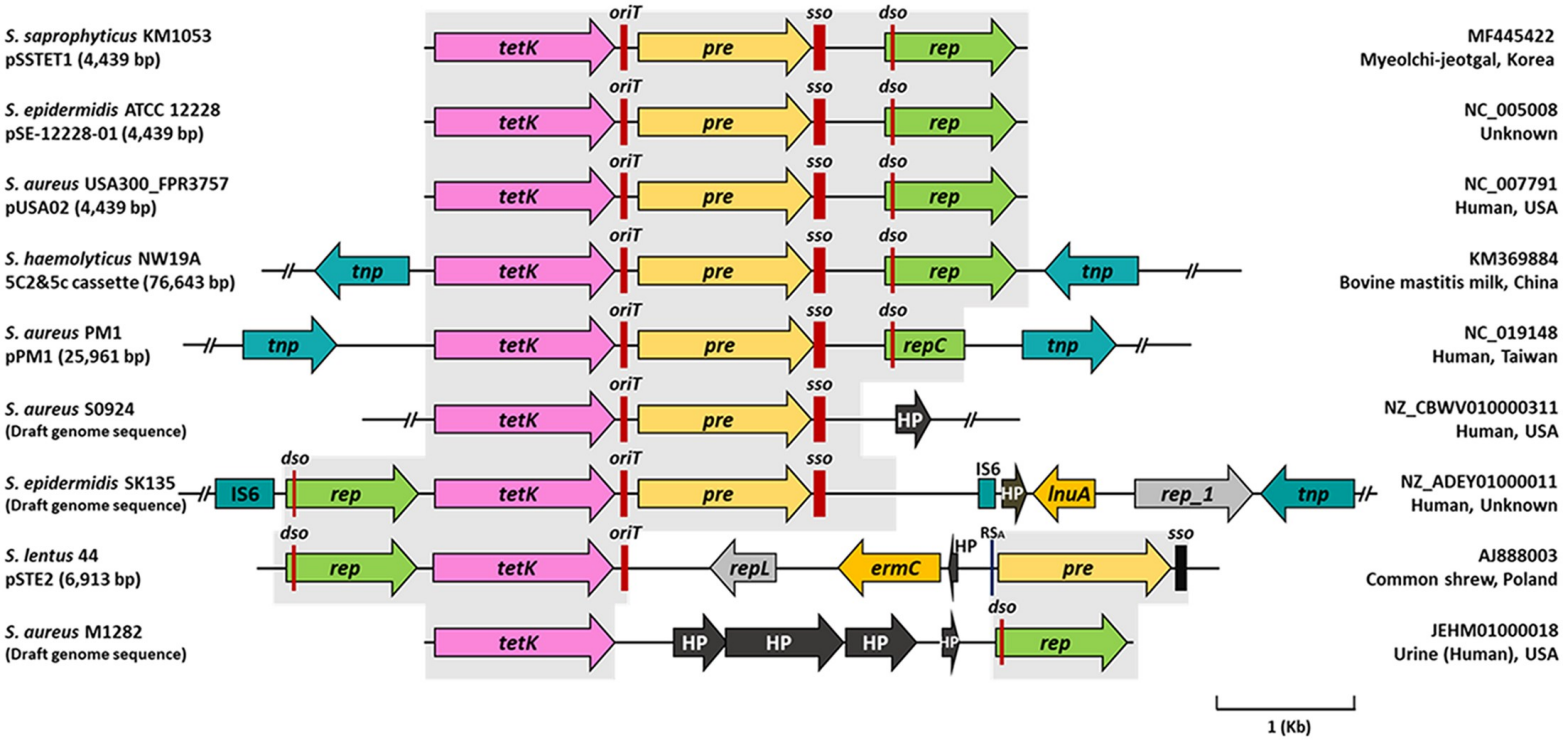
*tetK* genes and their flanking regions found in *Staphylococcus* species. Shaded boxed regions possess over 99% nucleotide sequence identity with pSSTET1. All sequences of *dso*, *sso* containing RS_B_, and *oriT* containing RS_A_ identified in this study are indicated with red vertical lines. Abbreviations: *oriT*, origin of transfer; *sso*, single-stranded origin of replication; *dso*, double-stranded origin of replication; *tetK*, tetracycline resistance gene; *lnuA*, lincomycin resistance gene; *ermC*, erythromycin resistance gene; *pre*, plasmid recombination enzyme gene; *rep*, replication protein gene; *repC*, truncated replication protein gene; *rep_1*, replication protein gene; *repL*, plasmid replication protein gene; *tnp*, IS431 transposase gene; IS6, IS6 family transposase gene; and HP, hypothetical protein gene.

Sequence analysis revealed that pSSTET1 contains elements that are typical of plasmids that replicate via a rolling-circle mechanism: the entire replication protein gene (*rep*), a double-stranded origin of replication (*dso*), a single-stranded origin of replication (*sso*), and an origin of transfer (*oriT*), together with *tetK* and a plasmid recombination enzyme gene (*pre*) (Fig 1). pSSTET1 had two nucleotides that differed from *S*. *aureus* USA300_FPR3757 pUSA02. One polymorphic site (G1417A) resulted in an amino acid difference in the TetK protein from the two organisms (S1 Fig). The other site (G3276A) may not cause a functional difference between the two plasmids. The nucleotide sequence of pSSTET1 has been deposited in the GenBank database under accession number MF445422.

### Characterization of pSSTET1

pSSTET1 contains three open reading frames corresponding to *tetK*, *pre*, and *rep*, which are transcribed in the same direction (Fig 1). The putative *tetK* gene encoded on pSSTET1 produces a protein of 430 amino acids, which exhibits 99.8% and 61.2% sequence identity with TetK from *S*. *aureus* USA300_FPR3757 pUSA02 [22] and TetL from *Lactobacillus sakei* (accession number: WP_012290101.1), respectively. TetK is recognized as an efflux protein that extrudes tetracycline. The *tetK* gene of pSSTET1 might confer tetracycline resistance to strain KM1053.

The *pre* gene of pSSTET1 encodes a putative plasmid recombination enzyme of 413 amino acids. Pre has been detected in Gram-positive bacteria where it has been shown to mediate recombination with a co-resident plasmid and conjugative plasmid transfer through the interaction of Pre and *oriT*, which contains the recombination site A (RS_A_) [23, 24]. RS_A_ is a recognition site of Pre and harbors a nick site in the sequence (5′-AAATAAGTCTAGTGTGTTAGACTT-3′). pSSTET1 has a RS_A_ site at nucleotides 1548–1571 within its *oriT* and the same structure has been identified from other staphylococcal plasmids (Fig 1 and S1 Fig). We assume that the gene organization in *Staphylococcus lentus* 44 pSTE2 and *S*. *aureus* M1282 might be generated by recombination events at RS_A_ sites (Fig 1). The heterologous cointegrates found in *S*. *lentus* 44 pSTE2 and *S*. *aureus* M1282 have been suggested to function as intermediates in the evolution of larger plasmids carrying multiple antibiotic resistance genes [25]. The emergence of multiple antibiotic-resistant plasmids as well as *tetK*-integrated chromosomes is expected.

The putative *rep* product comprises 314 amino acids and exhibits 100%, 99.7%, and 99.4% sequence identity with the Rep_trans proteins encoded on *S*. *aureus* USA300_FPR3757 pUSA02 [22], *Staphylococcus epidermidis* ATCC 12228 pSE-12228-01 [26], and *Staphylococcus haemolyticus* NW19A 5C2&5c cassette [27], respectively (Fig 1). Rep_trans protein, a replication initiation factor involved in rolling-circle replication, has been detected in various Gram-negative and positive bacterial plasmids and has several conserved protein motifs [28, 29]. Car and colleagues [30] suggested that the conserved motifs of the Rep_trans protein were: R140, D142, A144, D146, R212, and E214. The same active sites were found in the putative Rep_trans protein of pSSTET1 and two additional conserved motifs (G262 and T270) generally found in the Rep protein were also detected in the pSSTET1 Rep_trans protein (S1 Fig). Typically, the Rep protein including the Rep_trans protein is reported to recognize the *dso*-containing nick site [31] and pSSTET1 possesses a *dso* site (positions: 3456–3479) within the open reading frame of *rep* (positions: 3388–4332). Also, pSSTET1 possesses a recombination site B sequence (RS_B_) at nucleotides 2870–2884 (5′-TTTATGCCGAGAAAA-3′) and a 6-bp consensus sequence (CS-6) at nucleotides 2923–2928 (5′-TAGCGA-3′), which are the typical conserved sequences of *sso* [32]. The *sso* gene is known to have several inverted repeats that can generate stem-loop structures, which are known to be important in lagging strand initiation [33]. We detected two putative hairpin structures at positions 2857–2934, when an RNA secondary structure prediction program was employed (http://rna.urmc.rochester.edu). We observed a counter-transcribed RNA (ctRNA) sequence in pSSTET1 (3289–3418) that was located upstream of *rep* and was transcribed in the opposite direction. The ctRNA from the ctRNA sequence may regulate *rep* transcription by binding to its paired sequence within the promoter sequence of *rep* [34]. A putative promoter for ctRNA that consists of a ˗35 region (5′-TTGAAT-3′, 3413–3418) and a ˗10 region (5′-TATACA-3′, 3389–3394) is also present.

### Horizontal transfer of pSSTET1 by conjugation

Interspecific transfer of pSSTET1 was investigated by mating strains *S*. *saprophyticus* KM1053 and *S*. *equorum* KM1031, and by selecting for lincomycin resistance conferred by *lnuA*. Transconjugants showing phenotypic lincomycin and tetracycline resistance were detected at a frequency of 2.8 × 10^−6^ (Table 2). The plasmid was also successfully transferred to *E*. *faecalis* OG1RF, at frequencies of 1.2 × 10^−5^. However, pSSTET1 was not transferred into *S*. *equorum* KS1039 and *S*. *aureus* USA300 LAC.

**Table 2:** *In vitro* transfer of the *tetK* gene from *S*. *saprophyticus* to Gram-positive recipient strains.

Mating organism		Cell count (cfu/ml)			Transfer rate (T/R)	MIC of transconjugant	
Donor strain	Recipient strain	Donor	Recipient	Transconjugant^a^		Tet	Lin
*S*. *saprophyticus* KM1053	*S*. *equorum* KM1031	5.2 × 10^8^	3.4 × 10^8^	9.5 × 10^2^	2.8 × 10^−6^	>32	>512
*S*. *saprophyticus* KM1053	*S*. *equorum* KS1039	2.5 × 10^9^	9.4 × 10^7^	–			
*S*. *saprophyticus* KM1053	*S*. *aureus* USA300 LAC	1.6 × 10^9^	7.3 × 10^8^	–			
*S*. *saprophyticus* KM1053	*E*. *faecalis* OG1RF	5.0 × 10^8^	1.4 × 10^8^	5.8 × 10^3^	1.2 × 10^−5^	>32	>128

Cell counts were repeated three times independently and the mean values of the replicates are presented.

^a^ Transconjugants were confirmed by phenotypic resistance and 16S rRNA gene sequence analysis.

R, recipient; T, transconjugant.

The MIC for tetracycline of the donor strain *S*. *saprophyticus* KM1053 harboring pSSTET1 was 32 mg/l (Table 1). The *S*. *equorum* KM1031 and *E*. *faecalis* OG1RF transconjugants exhibited similar MICs to tetracycline (Table 2). Transconjugants exhibited lincomycin resistance, while the value was not the same as that of the recipients. The same phenomenon has been reported in previous studies that showed variation in the antibiotic susceptibility of transconjugants [35, 36]. Antibiotic susceptibility as well as the plasmid transfer ratio might be recipient strain-dependent characteristics.

### Segregational and structural stability of pSSTET1

The segregational and structural stability of pSSTET1 in donor and transconjugant strains was examined to validate the stability of the pSSTET1 replication system in wild-type hosts. The donor stain and transconjugant strains of *S*. *equorum* KM1031 and *E*. *faecalis* OG1RF exhibited tetracycline resistance after 100 generations in the absence of tetracycline. Importantly, the plasmid profile of the donor strain after 100 generations was the same as the original profile, without any obvious alterations in size for any of the plasmids examined and pSSTET1 was amplified from the donor and transconjugant strains (S2 Fig).

## Discussion

In the current study, plasmid pSSTET1 carrying the *tetK* gene was identified from *S*. *saprophyticus* isolated from fermented seafood. The plasmid harbors three genes, in the order *tetK*, *pre*, and *rep*, and the same gene organization is also identified in *Staphylococcus* spp. of human and animal origin (Fig 1). Before our identification of pSSTET1, the same gene organization has been identified from coagulase-positive *S*. *aureus* as well as CNS including *S*. *epidermidis*, *Staphylococcus hemolyticus*, and *S*. *lentus*. Although CNS are ubiquitously distributed in a vast array of natural origin, these CNS species have been mainly detected in skin and mucous membranes of mammals [37–40]. Among the three species, *S*. *epidermidis* and *S*. *haemolyticus* represent the major nosocomial pathogens as typical opportunists. While, *S*. *saprophyticus* has been frequently identified from fermented foods together with *S*. *equorum*, *Staphylococcus succinus*, and *Staphylococcus xylosus* [41]. In this context, CNS species of human and animal origin have higher potential chances to be exposed to antibiotics than those from foods. The advent of a food-originated *S*. *saprophyticus* harboring pSSTET1 insinuates the plasmid can be transferred in the absence of tetracycline exposure and our conjugal transfer experiment proved it.

The advent of three genes in pSSTET1 is not restricted to plasmids (Fig 1). *S*. *aureus* PM1 pPM1 and the *S*. *haemolyticus* NW19A 5C2&5c cassette have the same genetic organization of pSSTET1 being located within two transposases possessing the insertion sequence IS431. IS431, a well-known mobile genetic element found in *S*. *aureus*, is 782 bp long (IS431mec) and contains a putative transposase gene and 14- to 22-bp terminal inverted repeats [42, 43]. IS431 was reported to transfer a gene(s) or entire plasmid into other replicons or chromosomes. It has been reported to contribute to the insertion of antibiotic resistance genes into chromosomal DNA, as well as the deletion of antibiotic resistance genes from chromosomal DNA [44]. In addition, Liu and coworkers suggested that IS431 may facilitate the dissemination of antibiotic resistance genes [45]. The advent of a large plasmid pPM1 and chromosomal DNA possessing the three genes of pSSTET1 together with transposases raised a question about the origin of pSSTET1 in strain KM1053. We performed PCR using primer sets that can amplify IS431 and *tetK* simultaneously to detect IS431-mediated *tetK* genes in the chromosome of strain KM1053; however, the PCR amplicon was not obtained. Strain KM1053 might acquire pSSTET1 by an encounter with a donor under specific environmental pressure and can transfer pSSTET1 into other bacteria. Considering that *S*. *aureus* USA300_FPR3757 harboring pUSA02, a plasmid with the same structure as pSSTET1, was isolated from the USA, which is geographically distant, indicates that small plasmids with a similar structure to pSSTET1 are widely distributed and may be transferred into other bacteria.

*In vitro* transfer of pSSTET1 into a *S*. *equorum* strain provided evidence of the spread of plasmids with the pSSTET1 structure between *Staphylococcus* species in the same niche. However, unsuccessful transformation of *S*. *equorum* KS1039 and *S*. *aureus* USA300 LAC insinuates that the horizontal transfer of pSSTET1 to *Staphylococcus* species is conditional. Clinical *S*. *aureus* isolates are reputedly difficult to manipulate genetically and horizontal gene transfer is blocked by their restriction-modification system [46]. In the case of *S*. *equorum* KS1039, the CRISPR/Cas system, that prevents the uptake of foreign DNA, was identified in its genome [47]. Our plasmid transfer results confirmed the limited gene transfer to pathogenic *S*. *aureus*. This may ease concerns over the spread of antibiotic resistance from food fermentation starter cultures to pathogenic bacteria and confirms that the presence of transferable genes is not always linked with transferability. Notably, plasmids with a pSSTET1-type structure have only been found in staphylococci, while the possession of *tetK* in *Lactobacillus fermentum* and *Pediococcus pentosaceus* isolates from fermented foods has been reported [48]. The successful transfer of pSSTET1 into *E*. *faecalis* OG1RF further confirms the detection of pSSTET1-type plasmids in lactic acid bacteria. The transferability of pSSTET1 into lactic acid bacteria highlights the potential for further spread of tetracycline resistance from food to humans and the associated risk to human health. Thus, a better understanding of the molecular basis underlying this gene transfer mechanism is required in successive research to prevent the spread of pSSTET1-type plasmid. As of yet, the exact mechanism underlying pSSTET1 transfer remains unknown and it cannot be explained by plasmid sequence analysis, as conjugative transfer elements such as the relaxase gene have not been found. While, the *pre* gene detected on pSSTET1 was reported to encode a protein with a relaxase function involved in mobilization through the identification of RS_A_ in *oriT* [49]. In this context, we assume that the *pre* gene could be involved in the transfer of antibiotic resistance genes into other strains.

The emergence of antibiotic-resistant bacteria in fermented foods is a global concern. Further studies are required to fully understand the mechanisms responsible for the transfer of antibiotic resistance genes between bacteria. This study is the first to show the transfer of a mobile tetracycline resistance plasmid from food-originated *Staphylococcus* species to *Enterococcus* species and highlights the potential for tetracycline resistance gene transfer from *Staphylococcus* to the human commensal microbiota through food consumption. This study also confirmed that an antibiotic resistance gene in a mobile element is one of the important criteria in the selection and maintenance of starter cultures.

## Supporting information

S1 Fig Nucleotide sequence of pSSTET1. All elements involved in the origin for transfer (*oriT*), the double-stranded origin of replication (*dso*), and the single-stranded origin of replication (*sso*) region are shown in blue. The recombination site A sequence (RSA), recombination site B sequence (RSB), and a 6-bp consensus sequence (CS-6) are shown as black boxes. The nick sites are indicated as blue vertical arrowheads. Putative promoter regions of the *rep* and ctRNA genes are highlighted in red. The putative ctRNA stem-loop structure and inverted repeat sequences are indicated as differently-colored horizontal arrows. The ribosome binding sites are indicated in bold. The conserved amino acids of the putative Rep_trans protein, R, D, A, D, R, E G and T, are shown in bold red letters.
(DOCX)

S2 Fig PCR amplification of pSSTET1 in donor and transconjugant strains for 100 generations. Strains: 1, *S*. *saprophyticus* KM1053; 2, a transconjugant of *S*. *equorum* KM1031; a transconjugant of *E*. *faecalis* OG1RF.
(DOCX)
