# Supplementary material for: Transfer of a mobile Staphylococcus saprophyticus plasmid isolated from fermented seafood that confers tetracycline resistance
Source: PLoS One. 2019 Feb 28;14(2):e0213289. doi: 10.1371/journal.pone.0213289 (PMC6395029; doi:10.1371/journal.pone.0213289)
Supplement: S1 Fig — All elements involved in the origin for transfer (oriT), the double-stranded origin of replication (dso), and the single-stranded origin of replication (sso) region are shown in blue. The recombination site A sequence (RSA), recombination site B sequence (RSB), and a 6-bp consensus sequence (CS-6) are shown as black boxes. The nick sites are indicated as blue vertical arrowheads. Putative promoter regions of the rep and ctRNA genes are highlighted in red. The putative ctRNA stem-loop structure and inverted repeat sequences are indicated as differently-colored horizontal arrows. The ribosome binding sites are indicated in bold. The conserved amino acids of the putative Rep_trans protein, R, D, A, D, R, E G and T, are shown in bold red letters. (DOCX) [file pone.0213289.s001.docx]

| TGAAAGAATAATTTACCCCTATAAACTTTAGTCACCTCAAGTAAAGAGGTAAAATTGTTTAGTTTATATAAAAAATTTAAAGGT | 84 |
| --- | --- |
|  |  |
| TTGTTTTATAGCGTTTTATTTTGGCTTTGTATTCTTTCATTTTTTAGTGTATTAAATGAAATGGTTTTAAATGTTTCTTTACCT | 168 |
| ------------------**TetK** >>M--V--L--N--V--S--L--P |  |
| GATATTGCAAATCATTTTAATACTACTCCTGGAATTACAAACTGGGTAAACACTGCATATATGTTAACTTTTTCGATAGGAACA | 252 |
| D--I--A--N--H--F--N--T--T--P--G--I--T--N--W--V--N--T--A--Y--M--L--T--F--S--I--G--T-- |  |
| GCAGTATATGGAAAATTATCTGATTATATAAATATAAAAAAATTGTTAATTATTGGTATTAGTTTGAGCTGTCTTGGTTCATTG | 336 |
| A--V--Y--G--K--L--S--D--Y--I--N--I--K--K--L--L--I--I--G--I--S--L--S--C--L--G--S--L-- |  |
| ATTGCTTTTATTGGTCACAATCACTTTTTTATTTTGATTTTTGGTAGGTTAGTACAAGGAGTAGGATCTGCTGCATTCCCTTCA | 420 |
| I--A--F--I--G--H--N--H--F--F--I--L--I--F--G--R--L--V--Q--G--V--G--S--A--A--F--P--S-- |  |
| CTGATTATGGTGGTTGTAGCTAGAAATATTACAAGAAAAAAACAAGGCAAAGCCTTTGGTTTTATAGGATCAATTGTAGCTTTA | 504 |
| L--I--M--V--V--V--A--R--N--I--T--R--K--K--Q--G--K--A--F--G--F--I--G--S--I--V--A--L-- |  |
| GGTGAAGGGTTAGGTCCTTCAATAGGGGGAATAATAGCACATTATATTCATTGGTCTTACCTACTTATACTTCCTATGATTACA | 588 |
| G--E--G--L--G--P--S--I--G--G--I--I--A--H--Y--I--H--W--S--Y--L--L--I--L--P--M--I--T-- |  |
| ATAGTAACTATACCTTTTCTTATTAAAGTAATGGTACCTGGTAAATCAACAAAAAATACATTAGATATCGTAGGTATTGTTTTA | 672 |
| I--V--T--I--P--F--L--I--K--V--M--V--P--G--K--S--T--K--N--T--L--D--I--V--G--I--V--L-- |  |
| ATGTCTATAAGTATTATATGTTTTATGTTATTTACGACAAATTATAATTGGACTTTTTTAATACTCTTCACAATCTTTTTTGTG | 756 |
| M--S--I--S--I--I--C--F--M--L--F--T--T--N--Y--N--W--T--F--L--I--L--F--T--I--F--F--V-- |  |
| ATTTTTATTAAACATATTTCAAGAGTTTCTAACCCTTTTATTAATCCTAAACTAGGGAAAAACATTCCGTTTATGCTTGGTTTG | 840 |
| I--F--I--K--H--I--S--R--V--S--N--P--F--I--N--P--K--L--G--K--N--I--P--F--M--L--G--L-- |  |
| TTTTCTGGTGGGCTAATATTTTCTATAGTAGCTGGTTTTATATCAATGGTGCCTTATATGATGAAAACTATTTATCATGTAAAT | 924 |
| F--S--G--G--L--I--F--S--I--V--A--G--F--I--S--M--V--P--Y--M--M--K--T--I--Y--H--V--N-- |  |
| GTAGCGACAATAGGTAATAGTGTTATTTTTCCTGGAACCATGAGTGTTATTGTTTTTGGTTATTTTGGTGGTTTTTTAGTGGAT | 1008 |
| V--A--T--I--G--N--S--V--I--F--P--G--T--M--S--V--I--V--F--G--Y--F--G--G--F--L--V--D-- |  |
| AGAAAAGGATCATTATTTGTTTTTATTTTAGGATCATTGTCTATCTCTATAAGTTTTTTAACTATTGCATTTTTTGTTGAGTTT | 1092 |
| R--K--G--S--L--F--V--F--I--L--G--S--L--S--I--S--I--S--F--L--T--I--A--F--F--V--E--F-- |  |
| AGTATGTGGTTGACTACTTTTATGTTTATATTTGTTATGGGCGGATTATCTTTTACTAAAACAGTTATATCAAAAATAGTATCA | 1176 |
| S--M--W--L--T--T--F--M--F--I--F--V--M--G--G--L--S--F--T--K--T--V--I--S--K--I--V--S-- |  |
| AGTAGTCTTTCTGAAGAAGAAGTTGCTTCTGGAATGAGTTTGCTAAATTTCACAAGTTTTTTATCAGAGGGAACAGGTATAGCA | 1260 |
| S--S--L--S--E--E--E--V--A--S--G--M--S--L--L--N--F--T--S--F--L--S--E--G--T--G--I--A-- |  |
| ATTGTAGGAGGTTTATTGTCACTACAATTGATTAATCGTAAACTAGTTCTGGAATTTATAAATTATTCTTCTGGAGTGTATAGT | 1344 |
| I--V--G--G--L--L--S--L--Q--L--I--N--R--K--L--V--L--E--F--I--N--Y--S--S--G--V--Y--S-- |  |
| AATATTCTTGTAGCCATGGCTATCCTTATTATTTTATGTTGTCTTTTGACGATTATTGTATTTAAACGTTCTGAAAAGCAGTTT | 1428 |
| N--I--L--V--A--M--A--I--L--I--I--L--C--C--L--L--T--I--I--V--F--K--R--S--E--K--Q--F-- |  |
| GAATAGTTATATTATATTTTGGTTTAGAACTATGAGTGGCTAGCATTTTGCCACTCATTTTTTGCGTTAGCAAAAACAGGTTTA | 1512 |
| E--. ▼ nick site |  |
| AGCCTCGCAGAGCACACGTATTAACGACTTATTAAAAATAAGTCTAGTGT**GT**TAGACTTAAACTATTAAATACACATGAAACCT | 1596 |
| RS_A_ *oriT* |  |
|  |  |
| TTGTGCTT**AGGAG**TGATTTTTATATGTCTTATTCCATTGTTAGAGTTTCAAAAGTTAAATCTGGAACAAATACAACGGGCATAC | 1680 |
| -----------------**Pre** >>M--S--Y--S--I--V--R--V--S--K--V--K--S--G--T--N--T--T--G--I--Q |  |
| AAAAACATGTTCAAAGAGAAAATAATAATTATGAAAATGAAGATATAGACCATAGTAAAACTTACTTAAATTATGATTTGGTAA | 1764 |
| --K--H--V--Q--R--E--N--N--N--Y--E--N--E--D--I--D--H--S--K--T--Y--L--N--Y--D--L--V--N |  |
| ATGCTAATAAACAGAATTTTAATAACTTGATTGATGAAAAAATCGAACAGAATTATACAGGCAAAAGAAAAATTAGAACAGACG | 1848 |
| --A--N--K--Q--N--F--N--N--L--I--D--E--K--I--E--Q--N--Y--T--G--K--R--K--I--R--T--D--A |  |
| CGATTAAACACATTGATGGTTTAATTACATCAGACAATGATTTCTTTGATAATCAAACGCCAGAAGATACAAAGCAGTTTTTTG | 1932 |
| --I--K--H--I--D--G--L--I--T--S--D--N--D--F--F--D--N--Q--T--P--E--D--T--K--Q--F--F--E |  |
| AATATGCTAAAGAGTTTTTAGAACAAGAATACGGTAAAGATAATTTATTATATGCAACAGTTCACATGGACGAAAAAACACCAC | 2016 |
| --Y--A--K--E--F--L--E--Q--E--Y--G--K--D--N--L--L--Y--A--T--V--H--M--D--E--K--T--P--H |  |
| ATATGCATTATGGCGTTGTTCCAATAACTGATGATGGTCGTTTAAGTGCTAAAGAAGTTGTAGGTAATAAAAAAGCTTTAACAG | 2100 |
| --M--H--Y--G--V--V--P--I--T--D--D--G--R--L--S--A--K--E--V--V--G--N--K--K--A--L--T--A |  |
| CGTTTCAAGATAGATTTAATGAGCATGTTAAACAACGAGGATATGATTTAGAACGTGGGCAATCAAGACAAGTAACAAATGCTA | 2184 |
| --F--Q--D--R--F--N--E--H--V--K--Q--R--G--Y--D--L--E--R--G--Q--S--R--Q--V--T--N--A--K |  |
| AACATGAGCAAATAAGTCAGTATAAACAAAAAACAGAATATCATAAGCAAGAATATGAACGTGAGAGCCAAAAAACAGACCATA | 2268 |
| --H--E--Q--I--S--Q--Y--K--Q--K--T--E--Y--H--K--Q--E--Y--E--R--E--S--Q--K--T--D--H--I |  |
| TAAAGCAAAAGAACGATAAATTAATGCAAGAGTACCAAAAATCGTTAAATACGCTTAAAAAGCCTATAAATGTTCCGTATGAGC | 2352 |
| --K--Q--K--N--D--K--L--M--Q--E--Y--Q--K--S--L--N--T--L--K--K--P--I--N--V--P--Y--E--Q |  |
| AAGAAACTGAAAAAGTAGGTGGTTTATTTAGCAAAGAAATACAAGAAACTGGAAATGTTGTAATAAGCCAAAAAGATTTCAATG | 2436 |
| --E--T--E--K--V--G--G--L--F--S--K--E--I--Q--E--T--G--N--V--V--I--S--Q--K--D--F--N--E |  |
| AATTTCAGAAACAGATAAAAGCTGCTCAAGATATTTCGGAAGATTACGAGTATATAAAGTCTGGTAGAGCCTTAGATGATAAAG | 2520 |
| --F--Q--K--Q--I--K--A--A--Q--D--I--S--E--D--Y--E--Y--I--K--S--G--R--A--L--D--D--K--D |  |
| ATAAGGAAATACGAGAGAAAGATGATTTATTAAATAAAGCAGTTGAGCGTATTGAAAACGCAGACGATAATTTTAACCAACTTT | 2604 |
| --K--E--I--R--E--K--D--D--L--L--N--K--A--V--E--R--I--E--N--A--D--D--N--F--N--Q--L--Y |  |
| ACGAAAATGCAAAGCCACTTAAAGAGAATATAGAAATAGCGTTAAAGCTTTTAAAAATCTTACTAAAAGAGTTAGAACGAGTTT | 2688 |
| --E--N--A--K--P--L--K--E--N--I--E--I--A--L--K--L--L--K--I--L--L--K--E--L--E--R--V--L |  |
| TAGGAAGAAATACCTTTGCGGAAAGAGTTAATAAGTTAACAGAAGATGAACCAAAACTAAATGGTTTAGCAGGAAACTTAGATA | 2772 |
| --G--R--N--T--F--A--E--R--V--N--K--L--T--E--D--E--P--K--L--N--G--L--A--G--N--L--D--K |  |
| AAAAAATGAATCCAGAATTATATTCAGAACAGGAACAGCAACAAGAACAACAAAAGAATCAAAAACGAGATAGAGGTATGCACT | 2856 |
| --K--M--N--P--E--L--Y--S--E--Q--E--Q--Q--Q--E--Q--Q--K--N--Q--K--R--D--R--G--M--H--L |  |
|  |  |
|  |  |
| TATAGAACATGCATTTATGCCGAGAAAACTTATTGGTTGGAATGGGCTATGTGTTAGCTAACTTGTTAGCGAGTTGGTTGGACT | 2940 |
|  |  |
| RS_B_ CS-6 **sso** |  |
| TGAATTGGGATTAATCCCAAGAAAGTACCAACTCAACAACACATAAAGCCCTGTAGGTTCCGACCAATAAGGAAATTGGAATAA | 3024 |
|  |  |
| AGCAATAAAAGGAGTTGAAGAAATGAAATTCAGAGAAGCCTTTGAGAATTTTATAACAAGTAAGTATGTACTTGGTGTTTTAGT | 3108 |
|  |  |
| AGTTTTAACTGTTTACCAGATAATACAAATGCTTAAATAAAAAAAGACTTGATCTGATTAGACCAAGTCTTTTGATAGTGTTAT | 3192 |
|  |  |
| ATTAATAACAAAATAAAAAGGAGTCGCTCACGCCCTGACCAAAGTTTGTGAACGACATCATTCAAAGAAAAAAACACTGAGTTG | 3276 |
|  |  |
| TTTTTATAATCTTGTATATTTAGATATTAAACGATATTTAAATATACATCAAGATATATATTTGGGTGAGCGATTCCTTAAACG | 3360 |
| (P_rep_)> -35 -10 |  |
| -10 -35 <(P_ctRNA_) |  |
| AAATTGAGATTA**AGGAG**TCGATTTTTTATGTATAAAAACAATCATGCAAATCATTCAAATCATTTGGAAAATCACGATTTAGAC | 3444 |
| ---------------------**Rep** >>M--Y--K--N--N--H--A--N--H--S--N--H--L--E--N--H--D--L--D-- |  |
| - Nick site |  |
| AATTTTTCTAAAACCGGCTACTCTAATAGCCGGTTGGACGCACATACTGTGTGCATATCTGATCCAAAATTAAGTTTTGATGCA | 3528 |
| *dso* |  |
| N--F--S--K--T--G--Y--S--N--S--R--L--D--A--H--T--V--C--I--S--D--P--K--L--S--F--D--A-- |  |
|  |  |
| ATGACGATCGTTGGAAATCTCAACCGAGACAACGCTCAAGCCCTTTCTAAATTTATGAGTGTAGAGCCCCAAATAAGACTTTGG | 3612 |
| M--T--I--V--G--N--L--N--R--D--N--A--Q--A--L--S--K--F--M--S--V--E--P--Q--I--R--L--W-- |  |
| GATATTCTTCAAACAAAGTTTAAAGCTAAAGCACTTCAAGAAAAAGTTTATATTGAATATGACAAAGTGAAAGCAGATAGTTGG | 3696 |
| D--I--L--Q--T--K--F--K--A--K--A--L--Q--E--K--V--Y--I--E--Y--D--K--V--K--A--D--S--W-- |  |
| GATAGACGTAATATGCGTATTGAATTTAATCCAAACAAACTTACACGAGATGAAATGATTTGGTTAAAACAAAATATAATAAGC | 3780 |
| D--R--R--N--M--R--I--E--F--N--P--N--K--L--T--R--D--E--M--I--W--L--K--Q--N--I--I--S-- |  |
| TACATGGAAGATGACGGTTTTACAAGATTAGATTTAGCCTTTGATTTTGAAGATGATTTGAGTGACTACTATGCAATGTCTGAT | 3864 |
| Y--M--E--D--D--G--F--T--**R**--L--**D**--L--**A**--F--**D**--F--E--D--D--L--S--D--Y--Y--A--M--S--D-- |  |
| AAAGCAGTTAAGAAAACTATTTTTTATGGTCGTAATGGTAAGCCAGAAACAAAATATTTTGGCGTGAGAGATAGTAATAGATTT | 3948 |
| K--A--V--K--K--T--I--F--Y--G--R--N--G--K--P--E--T--K--Y--F--G--V--R--D--S--N--R--F-- |  |
| ATTAGAATTTATAATAAAAAGAAAGAACGTAAAGATAATGCAGATGCTGAAGTTATGTCTGAACATTTATGGCGTGTAGAAATC | 4032 |
| I--R--I--Y--N--K--K--K--E--R--K--D--N--A--D--A--E--V--M--S--E--H--L--W--**R**--V--**E**--I-- |  |
| GAACTTAAAAGAGATATGGTGGATTACTGGAATGATTGCTTTAGTGATTTACATATCTTGCAACCAGATTGGAAAACTATCCAA | 4116 |
| E--L--K--R--D--M--V--D--Y--W--N--D--C--F--S--D--L--H--I--L--Q--P--D--W--K--T--I--Q-- |  |
| CGCACTGCGGATAGAGCAATAGTTTTTATGTTATTGAGTGATGAAGAAGAATGGGGAAAGCTTCACAGAAATTCTAGAACAAAA | 4200 |
| R--T--A--D--R--A--I--V--F--M--L--L--S--D--E--E--E--W--**G**--K--L--H--R--N--S--R--**T**--K-- |  |
| TATAAGAATTTGATAAAAGAAATTTCGCCAGTCGATTTAACGGACTTAATGAAATCGACTTTAAAAGCGAACGAAAAACAATTG | 4284 |
| Y--K--N--L--I--K--E--I--S--P--V--D--L--T--D--L--M--K--S--T--L--K--A--N--E--K--Q--L-- |  |
| CAAAAACAAATCGATTTTTGGCAACATGAATTTAAATTTTGGAAATAGTGTACATATTAATATTACTGAACAAAAATGATATAT | 4368 |
| Q--K--Q--I--D--F--W--Q--H--E--F--K--F--W--K--. |  |
| TTAAACTATTCTAATTTAGGAGGATTTTTTTATGAAGTGTCTATTTAAAAATTTGGGGAATTTATATGAGG | 4439 |
|  |  |

**S1 Fig. Nucleotide sequence of pSSTET1.** All elements involved in the origin for transfer (*oriT*), the double-stranded origin of replication (*dso*), and the single-stranded origin of replication (*sso*) region are shown in blue. The recombination site A sequence (RSA), recombination site B sequence (RSB), and a 6-bp consensus sequence (CS-6) are shown as black boxes. The nick sites are indicated as blue vertical arrowheads. Putative promoter regions of the *rep* and ctRNA genes are highlighted in red. The putative ctRNA stem-loop structure and inverted repeat sequences are indicated as differently-colored horizontal arrows. The ribosome binding sites are indicated in bold. The conserved amino acids of the putative Rep_trans protein, R, D, A, D, R, E G and T, are shown in bold red letters.
