# Supplementary material for: Transfer of a mobile Staphylococcus saprophyticus plasmid isolated from fermented seafood that confers tetracycline resistance
Source: PLoS One. 2019 Feb 28;14(2):e0213289. doi: 10.1371/journal.pone.0213289 (PMC6395029; doi:10.1371/journal.pone.0213289)
Supplement: S2 Fig — Strains: 1, S. saprophyticus KM1053; 2, a transconjugant of S. equorum KM1031; a transconjugant of E. faecalis OG1RF. (DOCX) [file pone.0213289.s002.docx]

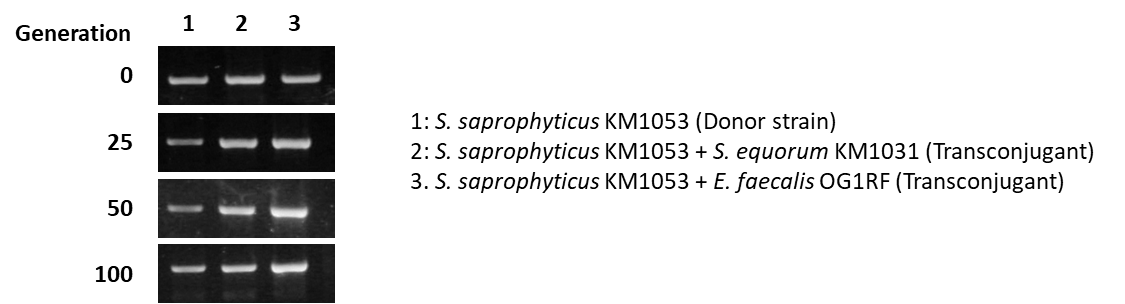


**S2 Fig. PCR amplification of pSSTET1 in donor and transconjugant strains for 100 generations.**

Strains: 1, *S. saprophyticus* KM1053; 2, a transconjugant of *S. equorum* KM1031; a transconjugant of *E. faecalis* OG1RF.
